# Loss of β-arrestin2 aggravated condylar cartilage degeneration at the early stage of temporomandibular joint osteoarthritis

**DOI:** 10.1186/s12891-024-07558-z

**Published:** 2024-06-06

**Authors:** Mengjiao Zhu, Ziwei Huang, Jing Qin, Jiafeng Jiang, Mingyue Fan

**Affiliations:** 1 Department of Orthodontics, Shanghai Xuhui District Dental Center, 500 Fenglin Road, Shanghai, China; 2 Department of Orthodontics, Nanjing Stomatological Hospital, Medical School of Nanjing University, 30 Central Road, Nanjing, China; 3 Department of Pediatric Dentistry, Shanghai Xuhui District Dental Center, 500 Fenglin Road, Shanghai, China

**Keywords:** β-arrestin2, Temporomandibular joint osteoarthritis, Cartilage degeneration, Inflammation, Apoptosis, Autophagy

## Abstract

**Objective:**

Temporomandibular joint osteoarthritis (TMJOA) is a chronic degenerative joint disorder characterized by extracellular matrix degeneration and inflammatory response of condylar cartilage. β-arrestin2 is an important regulator of inflammation response, while its role in TMJOA remains unknown. The objective of this study was to investigate the role of β-arrestin2 in the development of TMJOA at the early stage and the underlying mechanism.

**Methods:**

A unilateral anterior crossbite (UAC) model was established on eight-week-old wild-type (WT) and β-arrestin2 deficiency mice to simulate the progression of TMJOA. Hematoxylin-eosin (HE) staining and microcomputed tomography (micro-CT) analysis were used for histological and radiographic assessment. Immunohistochemistry was performed to detect the expression of inflammatory and degradative cytokines, as well as autophagy related factors. Terminal-deoxynucleotidyl transferase mediated nick end labeling (TUNEL) assay was carried out to assess chondrocyte apoptosis.

**Results:**

The loss of β-arrestin2 aggravated cartilage degeneration and subchondral bone destruction in the model of TMJOA at the early stage. Furthermore, in UAC groups, the expressions of degradative (Col-X) and inflammatory (TNF-α and IL-1β) factors in condylar cartilage were increased in β-arrestin2 null mice compared with WT mice. Moreover, the loss of β-arrestin2 promoted apoptosis and autophagic process of chondrocytes at the early stage of TMJOA.

**Conclusion:**

In conclusion, we demonstrated for the first time that β-arrestin2 plays a protective role in the development of TMJOA at the early stage, probably by inhibiting apoptosis and autophagic process of chondrocytes. Therefore, β-arrestin2 might be a potential therapeutic target for TMJOA, providing a new insight for the treatment of TMJOA at the early stage.

## Introduction

Temporomandibular joint disorders (TMDs) are a group of conditions that affect the temporomandibular joint (TMJ), masticatory muscles and associated structures [1]. TMD can be caused by aging, mechanical stress, psychological stress [2] or autoimmune disease like rheumatoid arthritis [3, 4] and SAPHO syndrome [5]. As one of the most severe subtypes of TMD, temporomandibular joint osteoarthritis (TMJOA) is a chronic degenerative joint disorder characterized by progressive destruction of articular cartilage and subchondral bone resorption [6]. The clinical manifestations of the disease are varied, including joint pain, restricted mouth opening, and joint cracking, which seriously affects people’s quality of life [7]. At present, the process of TMJOA is hindered mainly by symptomatic treatment and surgical treatment, but neither of them can completely repair the damaged TMJ and restore its function. Therefore, it is of great significance to explore related pathogenic genes and mechanisms for the treatment of TMJOA.

Although the explicit etiology of TMJOA is poorly understood, there are multiple risk factors, including estrogen, chondrocyte apoptosis, and excessive mechanical stress, contributing to the initiation and progression of TMJOA [8]. In addition, inflammation has been recognized as an important player in the pathogenesis of TMJOA [9]. It is widely accepted that TMJOA is a low-inflammatory arthritic condition [8]. Various proinflammatory cytokines, including interleukin IL-1β, IL-6, IL-8, and tumor necrosis factor (TNF) -a, were proved to be elevated in temporomandibular joint (TMJ) synovial fluid of TMJOA patients [9, 10]. Therefore, an effective control of inflammatory response in TMJOA might be an important therapeutic target for this disease.

β-arrestin2, which is encoded by *Arrb2* gene, is one of the scaffold proteins which involves in G-protein-coupled receptors (GPCRs) desensitization and down-regulation, and can transduce receptor signals independently of G protein [11, 12]. β-arrestin2 regulates multiple signaling pathways and is closely related to the inflammation involved diseases. It has been reported that β-arrestin2 plays an inhibitory role in the occurrence and development of arthritis, and the deletion of β-arrestin2 exhibits a more severe arthritis phenotype in the mouse collagen antibody-induced arthritis (CAIA) model [13]. Additionally, β-arrestin2 has been confirmed to negatively regulate the inflammatory response in other pathological process such as intestinal inflammation [14, 15] or liver injury [16]. This collected data suggests that up-regulation of β-arrestin2 might be a compensatory mechanism for maintaining homeostasis during inflammatory disease. Therefore, it is of great significance to clarify the role of β-arrestin2 in inflammatory diseases and further elucidate the mechanisms. However, due to the limited evidence, the role of β-arrestin2 in inflammatory diseases or TMJOA remains unknown.

Unilateral anterior crossbite (UAC) model, one of the mechanical loading models, is frequently used to induce typical pathological changes of TMJOA [17–19]. In the present study, the early-stage of TMJOA was established using a UAC model by disturbing the dental occlusion, and the effects of β-arrestin2 in the development of TMJOA were investigated. We demonstrated that the loss of β-arrestin2 aggravated the cartilage matrix degradation, cartilage degeneration and subchondral bone destruction at the early stage of TMJOA model. Additionally, we found that β-arrestin2 deficiency promoted inflammatory factors release, apoptosis and autophagic process of chondrocytes in the progression of TMJOA. In conclusion, we demonstrated for the first time that β-arrestin2 plays a protective role in the development of TMJOA at the early stage.

## Methods

### Animals models

All animal procedures were performed in accordance with relevant guidelines and approved by the Animal Welfare Committee of Shanghai Xuhui District Dental Center (China), and the approval number is [2021]4. The study was carried out in compliance with the ARRIVE guidelines (https://arriveguidelines.org).

Eight-week-old wild-type (WT) mice (GemPharmatech Co., Ltd., China) and *Arrb2* knockout (*Arrb2*^*−/−*^) mice were used in this study. All utilized mice were maintained on a C57/BL6 background. All mice were housed in a temperature (22–25^◦^C) and humidity-controlled (50% ± 10%) environment with a 12 h light/dark cycle. The total number of mice included in the present study was 72.

UAC operations were performed on eight-week-old WT and *Arrb2*^*−/−*^ mice as previously described [20]. Briefly, two suitable metal tubes made of a pinhead were attached to the left maxillary and mandibular incisors separately under deep anaesthesia with intraperitoneal injection of 1% pentobarbital. The tube for the mandibular incisors had a curved, 135°, labially inclined occlusal plate to create a crossbite relationship with the maxillary-tubed incisor [21]. Mice in the sham groups received the same procedure but without the attachment of metal tubes. All animals received the same diet and were fed cylindrically shaped pressed food pellets. Thus, mice were divided into four groups according to whether the mice received UAC or sham operation: WT (WT mice with sham operation), *Arrb2*^*−/−*^ (*Arrb2*^*−/−*^ mice with sham operation), WT + UAC (WT mice with UAC operation), *Arrb2*^*−/−*^+UAC (*Arrb2*^*−/−*^ mice with UAC operation).

### Tissue preparation and histological staining

All mice were sacrificed using 1% pentobarbital at the third week of operation. The TMJ samples from each subgroup were fixed in 4% paraformaldehyde at 4^◦^C overnight. After decalcification in 10% ethylenediaminetetraacetic acid (EDTA) for 1 month at 4^◦^C, the TMJ blocks were prepared for paraffin embedding, and sectioned through the TMJ sagittal plane to make 5-µm-thick serial sections. After deparaffinization and rehydration, hematoxylin-eosin (HE) staining, Safranin O and Fast Green staining (G1053, Servicebio, China) and TRAP staining (G1050, Servicebio, China) were carried out according to the manufacturer’s protocol. The thickness of the cartilage was measured in 3 squares located at the quartering points of the center third of the cartilage [22]. The mean of the values for the 3 squares in each section was used for further statistical analysis. The percentage of the Safranin O (SO) staining-positive area to the total area of cartilage in the central third of condylar cartilage in sagittal sections was calculated and expressed as mean ± standard deviation (SD) (*n* = 6).

### Immunohistochemistry (IHC) staining

The expression changes in matrix degradation and inflammation-related factors in condylar cartilage were investigated by IHC staining. The three-step avidin-biotin complex staining procedure was performed using type II collagen (Col-II) (sc-52,658, Santa Cruz, USA), type X collagen (Col-X) (BA2023, Boster, China), TNF-α (ab6671, Abcam, UK), IL-1β (ab283818, Abcam, UK), BECN1 (sc-48,341, Santa Cruz, USA) and LC3B-II (sc-271,625, Santa Cruz, USA) antibody. The IHC-positive cells were observed by Leica DFC490 system. The percentage of IHC-positive cells in the central third of condylar cartilage in sagittal sections was calculated by Adobe Photoshop CC. The average percentage of positive cells ± SD in each group was for further statistical analysis (*n* = 6).

### Microcomputed tomography (micro-CT) analysis

For micro-CT analysis, condylar heads isolated from mice were fixed in 70% ethanol and scanned using a micro-CT system (Scanco Medical, Switzerland) with a 10-µm scan resolution (10 μm per slice). A three-dimensional representative reconstruction image of the subchondral bone was obtained as a reference. Parameters such as bone volume fraction (BV/TV), trabecular number (Tb.N), trabecular separation (Tb.Sp) and trabecular thickness (Tb.Th) were measured (*n* = 6).

### Terminal -deoxynucleotidyl transferase mediated nick end labeling (TUNEL) assay

Chondrocyte apoptosis was assessed using a TUNEL assay with an in situ cell apoptosis detection kit (11,684,817,910, Roche, USA). Briefly, the sections were incubated with 20 µg/mL protease K for 20 min after deparaffinization and rehydration, followed by incubating with the prepared reaction mixture at 37 °C for 90 min. The sections were analyzed under a fluorescence microscope (BX-60, Olympus, Japan). The microscope had a 450–500 nm excitation filter, and the emission wavelength is 515–565 nm. The number of immunofluorescence-positive cells was counted and compared in condylar cartilage of each group (*n* = 6).

### Statistical analysis

We used IBM SPSS Statistics for Windows, Version 25.0 (IBM Corp., Armonk, NY, USA) for data sorting and analysis. All data are expressed as mean ± SD. The differences between the groups were analyzed by one-way analysis of variance. Six samples in each group were used for analysis. A P value of < 0.05 was considered statistically significant.

## Results

### Loss of β-arrestin2 aggravated the reduction of condylar cartilage thickness at the early stage of TMJOA

**Fig. 1:**
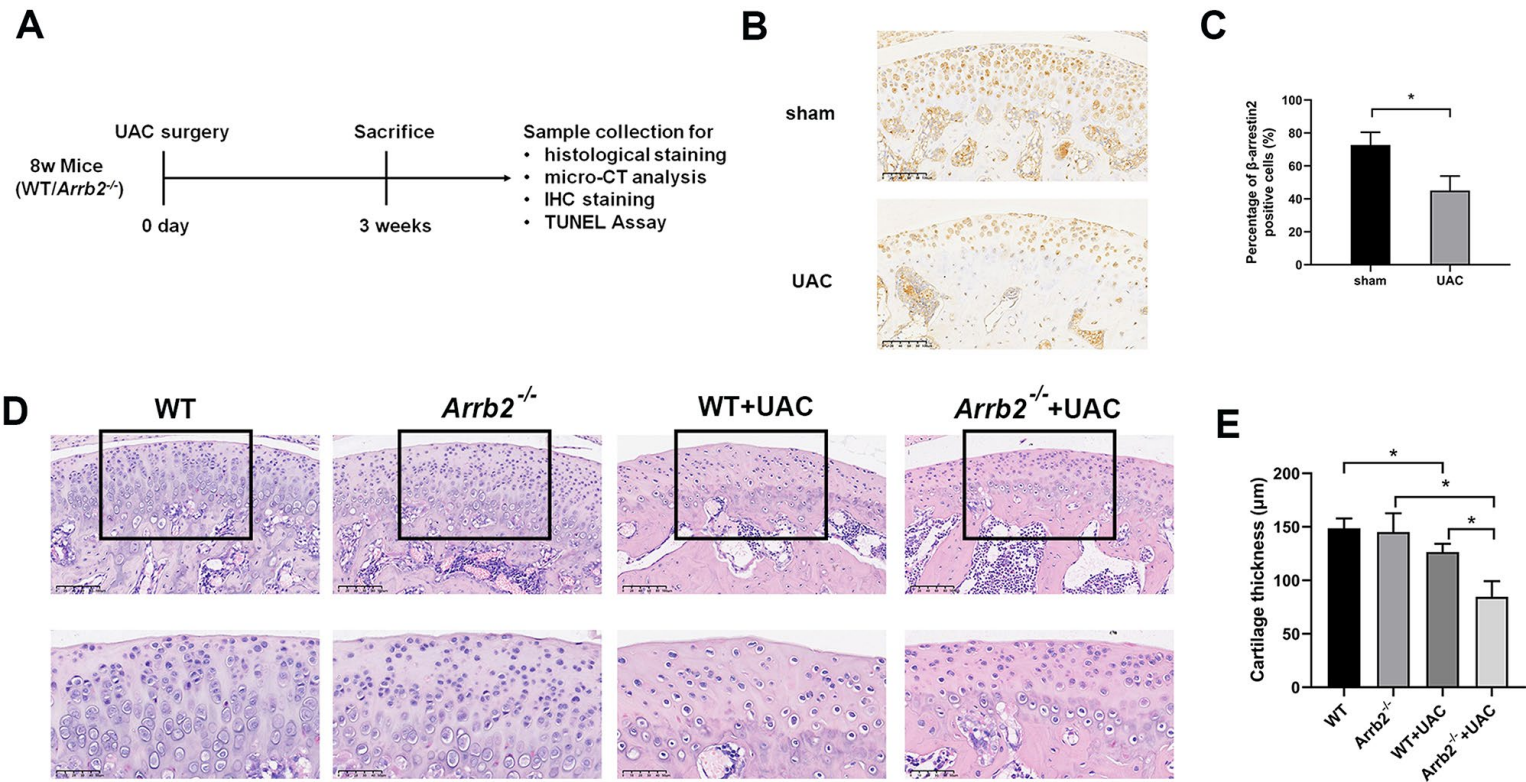
Effects of β-arrestin2 on condylar cartilage thickness at the early stage of TMJOA. (**A**) Schematic diagram of the animal experiment and main methodological steps. (**B**) Immunohistochemistry of β-arrestin2 in condylar cartilage of WT mice at 3 weeks of sham or UAC operation. (**C**) Quantitative analysis of positive cell rate of β-arrestin2 shown in 1 A (*n* = 6). (**D**) HE-stained sections of condylar cartilage at 3 weeks. (**E**) Quantification of cartilage thickness of the samples shown in 1 C (*n* = 6). Data were presented as mean *±* SD. *P < 0.05

A UAC model in mice for three weeks was adopted as previous reported to simulate a pathological change of an early stage of TMJOA (Fig. 1A) [1]. We first evaluated the expression change of the β-arrestin2 in mandibular condylar chondrocyte (MCC) at the early stage of TMJOA. Immunohistochemistry staining results showed that the expression of β-arrestin2 in MCC of UAC mice was significantly lower than that of control mice (Fig. 1B, C), which indicates that the expression of β-arrestin2 might be closely related to the development of TMJOA at the early stage.

To further elucidate the role that β-arrestin2 plays in the development of early-stage TMJOA, UAC operations were further performed on WT and *Arrb2*^*−/−*^ mice. Then mice were divided into four groups as follows: WT group (WT mice with sham operation), *Arrb2*^*−/−*^ group (*Arrb2*^*−/−*^ mice with sham operation), WT + UAC group (WT mice with UAC operation) and *Arrb2*^*−/−*^+UAC group (*Arrb2*^*−/−*^ mice with UAC operation).

HE results showed that *Arrb2*^*−/−*^ mice seem to have a more irregular chondrocyte arrangement and a lower chondrocyte density than that of WT group. However, the thickness of the cartilage in the *Arrb2*^*−/−*^ group was basically the same as those in WT group. After three weeks of UAC operation, WT + UAC and *Arrb2*^*−/−*^+UAC group exhibited reduced cartilage thickness, irregular chondrocyte arrangement and subchondral bone destruction compared with WT and *Arrb2*^*−/−*^ group respectively. Furthermore, the cartilage thickness of *Arrb2*^*−/−*^+UAC group was significantly decreased than that of WT + UAC group (Fig. 1D, E). The results indicated that the loss of β-arrestin2 aggravated the reduction of condylar cartilage thickness at the early stage of TMJOA.

### Loss of β-arrestin2 aggravated cartilage matrix degradation at the early stage of TMJOA

**Fig. 2:**
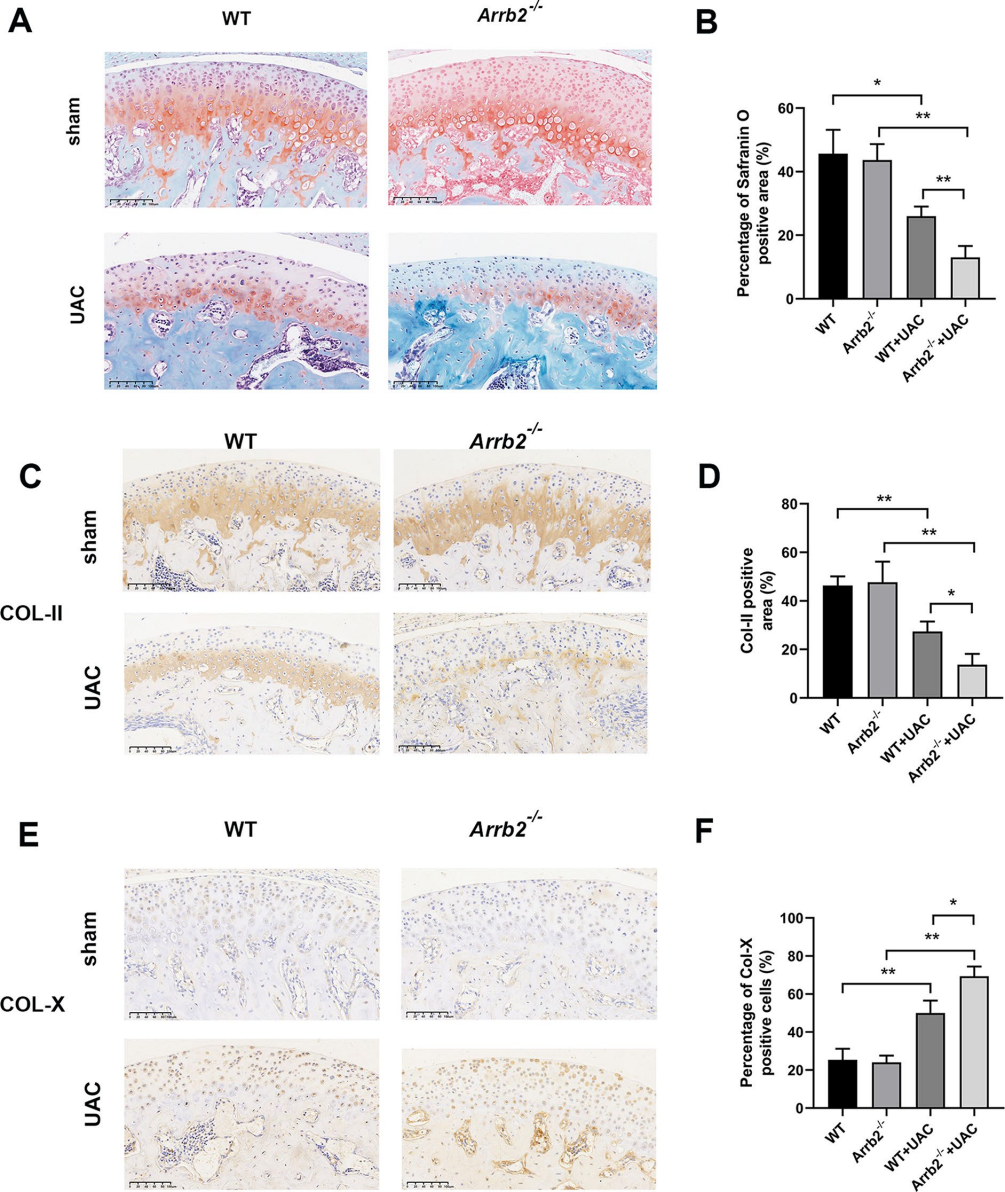
Loss of β-arrestin2 aggravated cartilage matrix degradation at the early stage of TMJOA. (**A**) Safranin O staining of condylar cartilage of different groups at 3 weeks. (**B**) Quantitative data of the percentage of Safranin O positive area (*n* = 6). (**C**) Immunohistochemistry of COL-II in condylar cartilage at 3 weeks. (**D**) Quantitative analysis of positive cell rate of COL-II shown in 2 C (*n* = 6). (**E**) Immunohistochemistry of COL-X in condylar cartilage at 3 weeks. (**F**) Quantitative analysis of positive cell rate of COL-X shown in 2 E (*n* = 6). Data were presented as mean *±* SD. *P < 0.05, **P < 0.01

Safranin O and Fast green staining was carried out to clarify the effect of β-arrestin2 on cartilage matrix. The results showed that SO-positive area showed no significant difference between WT and *Arrb2*^*−/−*^ group, but it was markedly decreased in WT + UAC and *Arrb2*^*−/−*^+UAC group, compared with WT and *Arrb2*^*−/−*^ group respectively. Additionally, SO-positive area was even more decreased in *Arrb2*^*−/−*^+UAC group compared with WT + UAC group (Fig. 2A, B). On the other hand, the immunohistochemistry results demonstrated that the expression of Col-II was decreased in WT + UAC and *Arrb2*^*−/−*^+UAC group compared with WT and *Arrb2*^*−/−*^ group respectively, and its expression was significantly decreased in *Arrb2*^*−/−*^+UAC group than that in WT + UAC group (Fig. 2C-D). In contrast, the expression of Col-X was increased in UAC groups, and its expression was increased in *Arrb2*^*−/−*^+UAC group than in WT + UAC group (Fig. 2E-F). Therefore, obvious condylar cartilage degeneration, characterized by degraded cartilage extracellular matrix was observed in UAC groups at early stage of TMJOA, and it was exacerbated in β-arrestin2 null mice. These results indicate that the loss of β-arrestin2 aggravated the condylar cartilage matrix degradation and cartilage degeneration at the early stage of TMJOA.

**Fig. 3:**
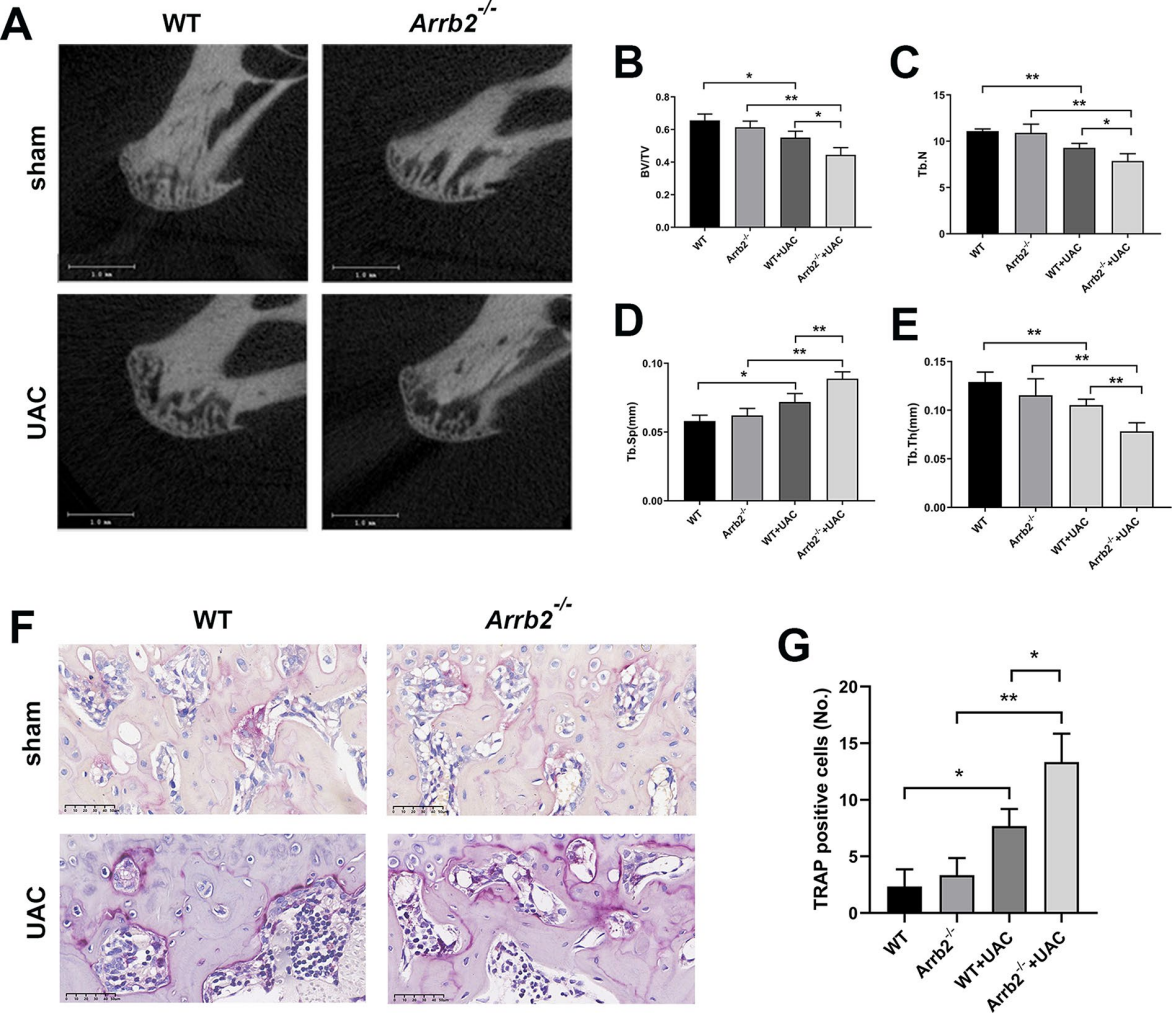
Loss of β-arrestin2 exacerbated subchondral bone destruction at the early stage of TMJOA. (**A**) Representative images showing trabecular architecture by micro-CT reconstruction in the subchondral bone at 3 weeks. (**B**) Quantitative analysis of BV/TV in the subchondral bone (*n* = 6). (**C**) Quantitative analysis of Tb.N in the subchondral bone (*n* = 6). (**D**) Quantitative analysis of Tb.Sp in the subchondral bone (*n* = 6). (**E**) Quantitative analysis of Tb.Th in the subchondral bone (*n* = 6). (**F**) Representative images of TRAP staining of the subchondral bone. (**G**) Quantitative analysis of TRAP positive cell numbers (*n* = 6). Data were presented as mean *±* SD. *P < 0.05, **P < 0.01

### Loss of β-arrestin2 exacerbated subchondral bone destruction at the early stage of TMJOA

According to the HE results above, three weeks of UAC operation induced apparent subchondral bone destruction of mandibular condylar. Therefore, micro-CT was carried out to observe the effect of β-arrestin2 on subchondral bone changes at the early stage of TMJOA. The results showed that compared with WT and *Arrb2*^*−/−*^ group, the bone volume fraction (BV/TV), trabecular number (Tb.N), and trabecular thickness (Tb.Th) were decreased while the trabecular separation (Tb.Sp) was increased in WT + UAC and *Arrb2*^*−/−*^+UAC group. Furthermore, those parameters showed no significant difference between WT and *Arrb2*^*−/−*^groups. However, BV/TV, Tb.N and Tb.Th were significantly decreased and Tb.Sp was increased in *Arrb2*^*−/−*^+UAC group, compared with WT + UAC group (Fig. 3A-E). TRAP staining was then performed to observe the histological changes of subchondral bone. Results showed that no significant difference was found in the number of TRAP positive cells between WT and *Arrb2*^*−/−*^groups. However, after UAC operation, the number of TRAP positive cells had a remarkable increase. Additionally, compared with WT + UAC group, the number of TRAP positive cells was significantly increased in *Arrb2*^*−/−*^+UAC group (Fig. 3F, G). These data demonstrated that β-arrestin2 deficiency aggravated subchondral bone destruction at the early stage of TMJOA.

**Fig. 4:**
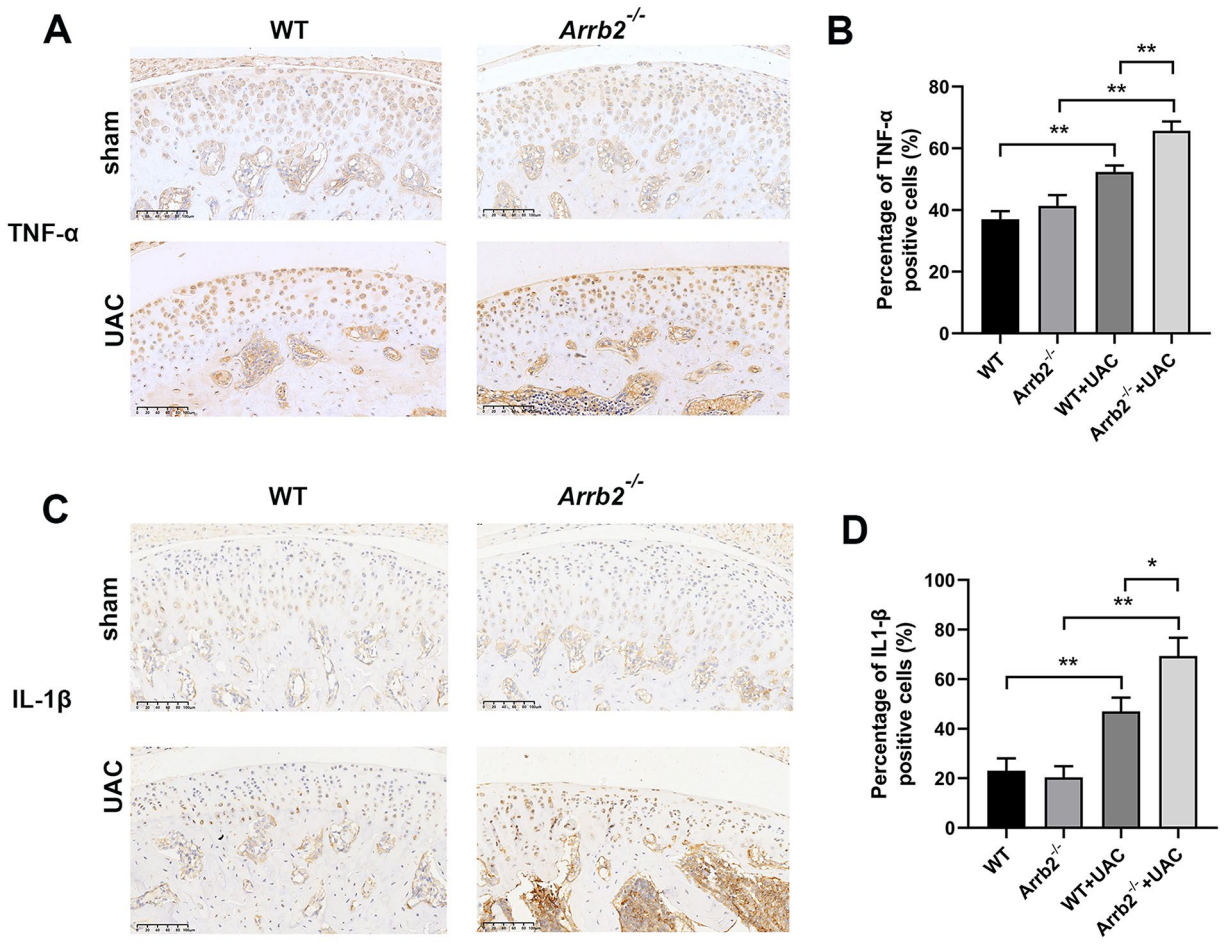
Loss of β-arrestin2 promotes chondrocyte inflammation at the early stage of TMJOA. (**A**) Immunohistochemistry of TNF-α in condylar cartilage at 3 weeks. (**B**) Quantitative analysis of positive cell rate of TNF-α shown in 4 A (*n* = 6). (**C**) Immunohistochemistry of IL-1β in condylar cartilage at 3 weeks. (**D**) Quantitative analysis of positive cell rate of IL-1β shown in 4 C (*n* = 6). Data were presented as mean *±* SD. *P < 0.05, **P < 0.01

### Loss of β-arrestin2 promotes chondrocyte inflammation at the early stage of TMJOA

To clarify what kind of effect does β-arrestin2 have on cartilage inflammation at the early stage of TMJOA, the expressions of inflammatory factors were detected. The immunohistochemistry results showed that there was no significant difference in TNF-α and IL-1β expressions between control and *Arrb2*^*−/−*^ group. After 3 weeks of UAC operation, both TNF-α and IL-1β expressions had a remarkable increase in UAC groups. In addition, their expressions were significantly higher in *Arrb2*^*−/−*^+UAC group than those in WT + UAC group (Fig. 4A-D). These data indicated that β-arrestin2 deficiency promotes the inflammation of chondrocytes at the early stage of TMJOA.

### Loss of β-arrestin2 increased chondrocyte apoptosis at the early stage of TMJOA

**Fig. 5:**
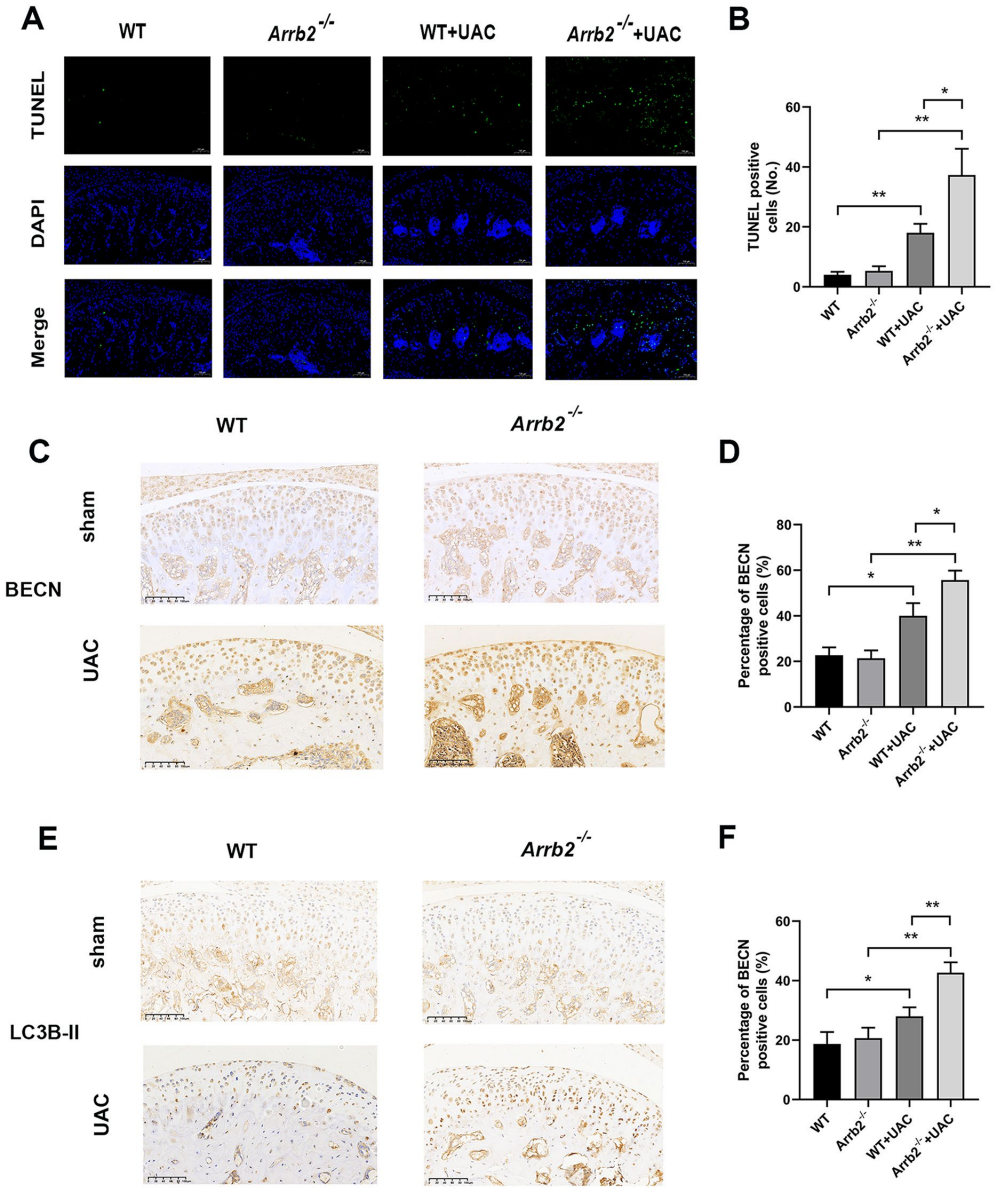
Loss of β-arrestin2 promoted apoptosis and autophagic process of chondrocytes at the early-stage TMJOA. (**A**) Representative images of TUNEL staining of the condylar cartilage. (**B**) Quantitative analysis of TUNEL positive cell numbers shown in 5 A (*n* = 6). (**C**) Immunohistochemistry of BECN in condylar cartilage at 3 weeks. (**D**) Quantitative analysis of positive cell rate of BECN shown in 5 C (*n* = 6). (**E**) Immunohistochemistry of LC3B-II in condylar cartilage at 3 weeks. (**F**) Quantitative analysis of positive cell rate of LC3B-II shown in 5E (*n* = 6). Data were presented as mean *±* SD. *P < 0.05, **P < 0.01

The progressive nature of TMJOA suggests the involvement of chondrocyte apoptosis in the cartilage degeneration [23]. Therefore, we next observed chondrocyte apoptosis using TUNEL staining. The results showed that chondrocyte apoptosis significantly increased in WT + UAC and *Arrb2*^*−/−*^+UAC group compared with WT and *Arrb2*^*−/−*^ group respectively. It was also increased in *Arrb2*^*−/−*^+UAC group than in WT + UAC group (Fig. 5A, B). The aforementioned results suggested that the lack of β-arrestin2 increased apoptosis of chondrocytes of TMJ condylar cartilage at early-stage TMJOA.

### Loss of β-arrestin2 promoted autophagic process of chondrocytes at the early stage of TMJOA

Given the important role of autophagy in maintaining cartilage homeostasis [24], we explored whether β-arrestin2 played the role in chondrocytes via autophagic mechanism. Immunohistochemistry staining results demonstrated that UAC operation promoted autophagic process by promoting the expressions of BECN1 and LC3B-II proteins in both WT and *Arrb2*^*−/−*^ groups. No significant difference was found between the two groups without UAC operations. However, the autophagic level was aggravated in *Arrb2*^*−/−*^+UAC group compared with WT + UAC group (Fig. 5C-F). Overall, the results suggested that the loss of β-arrestin2 accelerated cartilage degeneration probably by the abnormal activation of autophagic process.

## Discussion

With the increase of ages, both morphological and histological degenerative changes occur in the mandibular condylar cartilage [25]. TMJOA is a chronic degenerative joint disorder, and during this process, condylar flattening is the most common morphological change observed [26]. Inflammatory response and extracellular matrix degeneration are two typically pathological changes of TMJOA [2]. β-arrestin2 was reported to play a significant role in various inflammatory diseases [27]. However, the role of β-arrestin2 in TMJOA has not been described yet. In this study, we explored the effect of β-arrestin2 in cartilage degeneration of TMJOA at the early stage for the first time and preliminarily determined its potential mechanism. We confirmed that the loss of β-arrestin2 aggravated cartilage degeneration and subchondral bone destruction in the model of TMJOA at the early stage. Additionally, we found that β-arrestin2 deficiency increased the expression of inflammatory factors. Moreover, the loss of β-arrestin2 promoted apoptosis and autophagic process of chondrocytes in the progression of early-stage TMJOA. Overall, our evidences confirmed the protective role of β-arrestin2 in TMJOA probably by inhibiting the apoptosis and autophagic process of chondrocytes. This is the first known study which explores the role of β-arrestin2 in the progression of TMJOA.

In this study, typical TMJOA-like lesions in UAC groups were observed at 3 weeks, including decreased cartilage thickness and cartilage matrix degradation. The expressions of inflammatory and degradative factors were significantly increased in condylar cartilage of UAC groups. Thus, the early-stage TMJOA model was successfully established in UAC groups, which was consistent with previous researches [1, 28].

In addition, we found evidence that β-arrestin2 null mice were more sensitive to aberrant mechanical stress, including a more serious cartilage degeneration and subchondral bone destruction compared with control mice. This could be explained by the increased expression of inflammatory factors of chondrocytes in *Arrb2*^*−/−*^+UAC group. During the development of TMJOA, the increased secretion of the inflammatory cytokines, such as IL-1β, IL-6 or TNF-α, can induce destructive effect on chondrocytes and mesenchymal cells, including the decreased production of type II collagen and aggrecan, limiting chondrogenesis [29]. According to our study, the loss of β-arrestin2 might promote the secretion of inflammatory factors under the abnormal stress, which suggests that β-arrestin2 could be an anti-inflammatory factor at the early stage of TMJOA.

Programmed cell death, such as apoptosis and autophagy, plays an important role in TMJOA cartilage lesions [30]. Apoptosis provides space for neovascularization and is the source of abnormal cartilage mineralization, leading to the destruction of cartilage and subchondral bone of TMJ [31]. In the present study, we demonstrated that the apoptosis was induced and the autophagy process was activated in chondrocytes at the early stage of TMJOA, which is consistent with a previous research [23]. Additionally, we showed that in the absence of β-arrestin2, the apoptosis of chondrocytes was increased and the autophagic process was even more activated. Therefore, our results proved a protective role of β-arrestin2 in preventing apoptosis and autophagy, which is consistent with a previous study which demonstrated that the depletion of β-arrestin2 aggravates apoptosis of astrocytes stimulated by IL-6 [32]. In contrast, another study showing that β-arrestin2 depletion alleviated cell apoptosis by downregulating GRP78-ATF6-CHOP apoptosis signaling [33], which is contrary to our findings. Therefore, β-arrestin2 seems to play different roles in different pathological process, probably by interacting with different signaling molecules.

The multifaceted roles of β-arrestins in various diseases suggest that they may serve as promising therapeutic targets [34, 35]. For example, β-arrestin2 mediated extracellular matrix remodeling by inhibiting activating transcription factor 6 (ATF6) signaling, which ultimately protects periodontal tissues. Therefore, β-arrestin2 might be an effective therapeutic target of treating periodontitis [36]. Additionally, during the progression of rheumatoid arthritis, β2-adrenergic receptor agonist can prevent detrimental alterations in chondrocytes and support cartilage homeostasis by regulating the expression of arrestins in chondrocytes [37]. These examples illustrate ongoing research into the potential clinical applications of arrestins in various diseases, highlighting their importance in medical research and potential drug development. However, direct clinical applications or relevant drugs have not yet been achieved.

β-arrestin2 as one of the arrestins, was initially identified for its role in homologous desensitization and internalization of GPCRs. It is also a scaffolding protein and can transduce signaling by interacting with other signaling molecules independent of its role of mediating GPCR desensitization [38, 39]. It was recently reported that β-arrestin2 regulates immune response through a direct interaction with NF-κB, thus regulating NF-κB signaling [40]. Also, it is well established that NF-κB regulates the expression of inflammatory factors (including IL-6) [40]. Therefore, β-Arrestin2 might regulate the development of TMJOA through the NF-κB signal pathway. The focus of this paper does not fully clarify the detailed mechanism, which still need to be investigated in further research.

Finally, this study has several limitations. First, although we demonstrated that β-arrestin2 plays a protective role in the development of TMJOA at the early stage, probably by inhibiting apoptosis and autophagic process of chondrocytes, the detailed mechanism underlying this was not clarified. Additionally, in the present study, we mainly focus on the role of β-arrestin2 in early-stage TMJOA. However, its role in late-stage TMJOA is also of great significance. Therefore, further studies are needed to clarify the molecular mechanisms as well as the role of β-arrestin2 in late-stage TMJOA.

## Conclusions

In conclusion, our findings demonstrated for the first time that β-arrestin2 plays a protective role in the development of TMJOA at the early stage, probably by inhibiting apoptosis and autophagic process of chondrocytes. Therefore, β-arrestin2 might be a potential therapeutic target for TMJOA, providing a new insight for the treatment of TMJOA at the early stage.

## Data Availability

The datasets used and/or analysed during the current study are available from the corresponding author on reasonable request.

## Abbreviations

TMJOA: Temporomandibular Joint Osteoarthritis
UAC: Unilateral Anterior Crossbite
HE: Hematoxylin-Eosin
micro-CT: Microcomputed Tomography
TUNEL: Terminal-Deoxynucleotidyl Transferase Mediated Nick End Labeling
TMJ: Temporomandibular Joint
GPCRs: G-Protein-Coupled Receptors
CAIA: Collagen Antibody-Induced Arthritis
WT: Wild-Type
Arrb2^−/−^: Arrb2 Gene Knockout
EDTA: Ethylenediaminetetraacetic Acid
SO: Safranin O
IHC: Immunohistochemistry
SD: Standard Deviation
BV/TV: Bone Volume Fraction
Tb.Th: Trabecular Thickness
Tb.N: Trabecular Number
Tb.Sp: Trabecular Separation
MCC: Mandibular Condylar Chondrocyte
TNF-α: Tumor Necrosis Factor-α
IL-6: Interleukin-6
BECN1: Beclin1
LC3B-II: MAP1LC3B
TRAP: Tartrate Resistant Acid Phosphatase
IL-1β: Interleukin-1β
GRP78: Glucose-Regulated Protein 78
ATF6: Activating Transcription Factor 6
CHOP: The C/EBP Homologous Protein
NF-κB: Nuclear Factor Kappa-B
